# Development and validation of a risk score for predicting mortality after resection of primary hepatocellular carcinoma

**DOI:** 10.18632/aging.103360

**Published:** 2020-06-21

**Authors:** Xiang Zhou, Bin-Bin Cai, Xiang-Qing Hou, Xing-Kai Kang, Xiang-Xiang Xu, Wei-Ming Wang

**Affiliations:** 1 Key Laboratory of Diagnosis and Treatment of Severe Hepato-Pancreatic Diseases of Zhejiang Province, The First Affiliated Hospital of Wenzhou Medical University, Wenzhou, China; 2 Department of Hepatobiliary Surgery, The First Affiliated Hospital of Wenzhou Medical University, Wenzhou, Zhejiang, China; 3 Department of Preventive Medicine, School of Public Health and Management, Wenzhou Medical University, Wenzhou, Zhejiang, China

**Keywords:** PHCC, mortality, prognosis, X-tile, Hosmer-Lemeshow

## Abstract

Background: Primary hepatocellular carcinoma (PHCC) has a poor prognosis and high short-term mortality rate, even after resection. Thus, early diagnosis in PHCC cases can help improve quality of life via personalized management strategies.

Results: The risk score system (RSS) were classified as low risk (<5 points), medium risk (5–10 points), or high risk (>10 points). The areas under the receiver operating characteristic curves were 0.80 in the training cohort and 0.69 in the validation cohort, which indicated satisfactory prognostic performance. The Hosmer-Lemeshow goodness of fit test (*P*>0.05) revealed consistent performance in both groups. The concordance index (C-index: 0.663, 95% CI: 0.618–0.708) revealed excellent discrimination and good calibration in the validation cohort.

Conclusions: This simple RSS, which is based on clinical and laboratory data from patients undergoing resection of PHCC, might allow clinicians and medical staff to better manage PHCC.

Materials and Methods: A total of 672 PHCC cases were retrospectively obtained from the First Affiliated Hospital of Wenzhou Medical University between January 2007 and February 2015. Cox proportional hazard models were used to identify independent predictors of mortality. Kaplan-Meier curves and the log-rank test were used to examine the relationships between the prognostic factors and overall mortality.

## INTRODUCTION

In 2018, the International Agency for Research on Cancer (IARC) present an updated overview of the global cancer burden, which indicated that liver cancer has become the fourth leading cause of cancer-related death [1], an increase from its position as the sixth leading cause in 2012 [2]. Hepatocellular carcinoma (HCC) accounts for approximately 80% of primary liver cancer cases, with high-risk HCC areas (including China) having rapidly increasing HCC incidence and mortality rates, with the most prevalent risk factors being the hepatitis B virus in China and the hepatitis C virus in Japan [3]. Relative to transplantation or other treatment, resection based on the tumor’s extent is the preferred first-line treatment based on better survival outcomes, especially for early-stage HCC cases [4]. Therefore, the focus of recent HCC research has been concentrated on the effective management of surviving patients. Nevertheless, this approach requires an early evaluation of mortality risk at the resection, which guides the subsequent patient management decisions.

An increasing number of reports have focused on pathological or laboratory parameters that can predict the prognosis of various diseases and tumors [3, 5–7]. However, single factors are often ineffective for diagnosis and do not provide satisfactory results, which suggests that using multiple biomarkers can improve prognostic accuracy. For example, various prognostic tools have been developed to predict recurrence-free overall survival (OS) based on multiple significant risk factors [8–10]. Moreover, various risk factors have been found to predict primary HCC (PHCC)-related mortality, and several studies have shown that some internationally recognized and widely used systems can help predict the prognosis in PHCC cases [11–14]. Unfortunately, there is no universal and standardized scoring system for use in PHCC cases, as the existing staging and risk scoring systems (RSSs) have various limitations. Such as TNM (T: the extent of the primary tumor; N: regional lymph nodes; and M: distant metastases) system or the Child-Pugh-score, which only depends on tumor stage or impairment of liver function, thus can't explain the complexity of liver cancer in cirrhosis [15]. BCLC has been criticized for being too algorithmic to beneficial for clinical use [16]. Besides, The Okuda system has proven to be inadequate for patients with less advanced disease (early HCC) as well as is not bad but far from the excellent predictive ability [17].

Therefore, we attempted to overcome this limitation by constructing a simple and effective risk score system, which aimed to predict all-cause mortality after resection of PHCC.

## RESULTS

### Characteristics of the study population

This retrospective study identified 672 eligible patients between 2007 and 2015. Most patients were men (83.33%) and the median age was 57.0 years (IQR: 49.0–64.0 years). The patients were randomized 1:1 to the training cohort (336 patients) and the validation cohort (336 patients), with no significant differences detected in the cohorts’ baseline characteristics except the tumor size and platelet count (Supplementary Table 1). By August 1, 2018, we had identified 155 deaths in the training cohort (46.13%), with a mortality rate of 123.3/1,000 person-years (95% CI: 120.4–126.4). The validation cohort included 166 deaths (49.40%), with a mortality rate of 135.0/1,000 person-years (95% CI: 131.4–138.4). The average survival times were 3.74 years in the training cohort and 3.66 years in the validation cohort. The median follow-up time were 4.17 years in the training cohort and 4.01 years in the validation cohort.

### Prognostic factors in the training cohort

Table 1 shows the characteristics of the 336 patients in the training cohort (280 men and 56 women, median age: 57.0 years, IQR: 49.0–63.0 years). The univariate Cox regression models revealed that mortality was associated with LNM, tumor stage, satellite nodules, single/multiple tumors, PVTT, vascular infiltration, IATO, presence of ascites, tumor size, and values for PT, neutrophil count, albumin, TBIL, TC, AST, and γ-GTT. The multivariable Cox regression analyses revealed that mortality was independently predicted by higher tumor stage, multiple tumors, PVTT, IATO, greater tumor size, and elevated values for PT and AST.

**Table 1:** Population characteristics of the development cohort and crude/adjust association of potential prognostic determinants with death.

**Variables**	**All patients N,%**	**Live N,%**	**Dead N,%**	**P-value^¶^**	**Crude HR(95%CI)**	**Adjust HR(95%CI)**
Characteristic	336	181(53.87)	155(46.13)			
Woman,%	56(33.07)	33(58.9)	23(41.1)	0.701	0.917(0.589,1.428)	
Drink,%	151(33.07)	77(51.0)	74(49.0)	0.567	1.096(0.800,1.503)	
Vascular invasion,%	37(11.01)	20(54.1)	17(45.9)	0.588	1.150(0.694,1.903)	
LNM,%	8(2.38)	2(25.0)	6(75.0)	0.013	2.813(1.242,6.371)	
Tumor stage (Grade3/4), %	82(24.40)	33(40.2)	49(59.8)	0.001	0.567(0.404,0.796)	1.600(1.118.2.290)
Peri-cancerous invasion,%	14(4.17)	7(50.0)	7(50.0)	0.466	1.326(0.621,2.830)	
Envelope,%	71(21.13)	33(46.5)	38(53.5)	0.062	1.417(0.983,2.044)	
Satellite nodules, %	23(6.85)	8(34.8)	15(65.2)	0.007	2.090(1.226,3.564)	
Multiple tumors, %	40(11.90)	11(27.5)	29(72.5)	0.001	2.211(1.475,3.314)	1.865(1.119,3.107)
PVTT,%	9(2.68)	0(0.0)	9(100.0)	0.001	5.916(2.990,11.707)	4.194(1.327.13.251)
Vascular infiltration,%	18(5.36)	3(16.7)	15(83.3)	0.001	4.035(2.352,6.923)	
IATO,%	20(5.95)	1(5.0)	19(95.0)	0.001	0.215(0.132,0.352)	2.987(1.662,5.368)
Liver cirrhosis,%	238(70.83)	129(54.2)	109(45.8)	0.695	0.933(0.661,1.318)	
Ascites fluid,%	52(15.48)	22(42.3)	30(57.7)	0.013	1.657(1.112,2.469)	
HBsAg (Positive), %	279(83.04)	145(52.0)	134(48.0)	0.090	1.490(0.940,2.360)	
AFP(≥400 ug/L),%	72(21.43)	36(50.0)	36(50.0)	0.282	1.227(0.845,1.782)	
Age	57.0(49.0,63.0)	57.0(49.0,63.0)	57.0(49.0,65.0)	0.771	1.002(0.987,1.017)	
BMI(Kg/m2),%						
<18.5	23(6.85)	12(52.2)	11(47.8)	Ref	Ref	
18.5~23.9	194(57.74)	96(49.5)	98(50.5)	0.886	1.047(0.561,1.953)	
>23.9	119(35.42)	73(61.3)	46(38.7)	0.400	0.754(0.390,1.456)	
Tumor size(cm)	3.5(2.5,5.0)	3.0(2.0,5.0)	4.0(3.0,7.0)	0.001	1.148(1.103,1.194)	1.103(1.045,1.164)
PT(s)	13.9(13.3,14.7)	13.8(13.3,14.4)	14.2(13.4,15.0)	0.001	1.278(1.132,1.442)	1.273(1.063,1.526)
Preoperative fibrinogen(g/L)	2.8(2.4,3.4)	2.8(2.4,3.3)	2.9(2.3,3.6)	0.296	1.090(0.928,1.280)	
Neutrophil count(x109/L)	3.2(2.4,4.2)	3.2(2.4,4.2)	3.1(2.3,4.2)	0.036	1.060(1.004,1.119)	
Mononuclear cell count(x109/L)	0.45(0.30,0.60)	0.5(0.3,0.6)	0.4(0.3,0.6)	0.130	1.587(0.872,2.887)	
Lymphocvte count(x109/L)	1.40(1.00,1.85)	1.5(1.1,1.8)	1.4(0.9,1.9)	0.349	0.881(0.676,1.148)	
Platelet count(x109/L)	136.0(94.5,171.0)	138.0(97.0,172.0)	135.0(84.0,168.0)	0.873	1.000(0.997,1.002)	
Albumin(g/L)	39.7(36.3,43.0)	40.5(37.3,43.8)	38.6(34.3,42.2)	0.001	0.935(0.911,0.960)	
TBIL(μmol/L)	11.0(8.0,16.0)	11.0(8.0,14.0)	12.0(9.0,19.0)	0.001	1.037(1.022,1.053)	
TC(mmol/l)	4.3(3.5,4.9)	4.3(3.6,4.9)	4.0(3.5,4.7)	0.036	0.840(0.713,0.988)	
ALT(U/L)	34.0(24.0,54.5)	32.0(23.0,43.0)	39.0(25.0,66.0)	0.062	1.002(1.000,1.004)	
AST(U/L)	40.5(30.0,68.0)	34.0(27.0,49.0)	50.0(33.0,120.0)	0.003	1.002(1.001,1.003)	1.002(1.000,1.003)
γ-GTT(U/L)	54.0(34.0,106.0)	48.0(30.0,99.0)	64.0(39.0,119.0)	0.045	1.001(1.000,1.003)	

**Abbreviations:** BMI: body mass index; PT: prothrombin time; TBIL: total bilirubin; TC: total cholesterol; ALT: alanine transaminase; AST: aspartate transaminase; γ-GT: γ-glutamyl transpeptidase; LNM: lymph node metastasis; PVTT: portal vein tumor thrombus; IATO: invasion of adjacent tissues or organs; HR: Hazard ratio; CI: Confidence incidence;

¶:P values are for the comparison of corresponding reference group with patients in Categorical variables (%), Univariate cox analysis as appropriate, for the other Continuity variables.

### X-tile analysis and survival analysis

**Figure 1:**
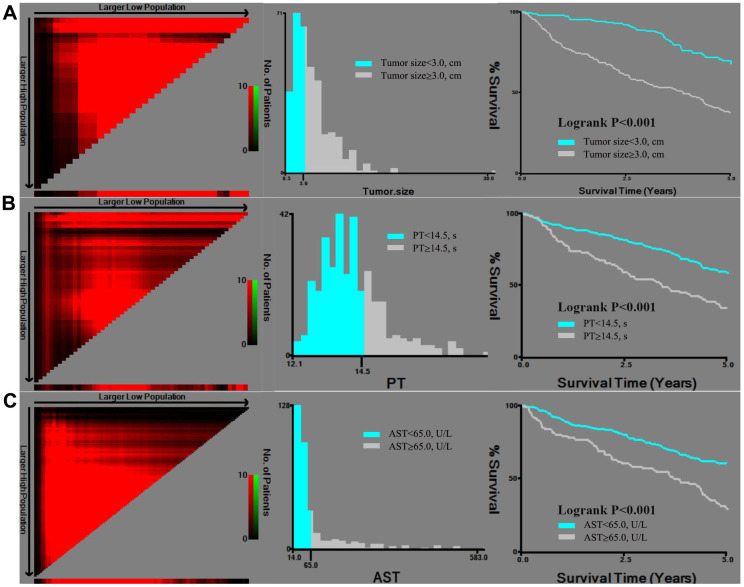
X-tile analysis of tumor size (**A**), PT (**B**), and AST (**C**), which were independently associated with mortality. Abbreviations: PT: prothrombin time; AST: aspartate transaminase.

**Figure 2:**
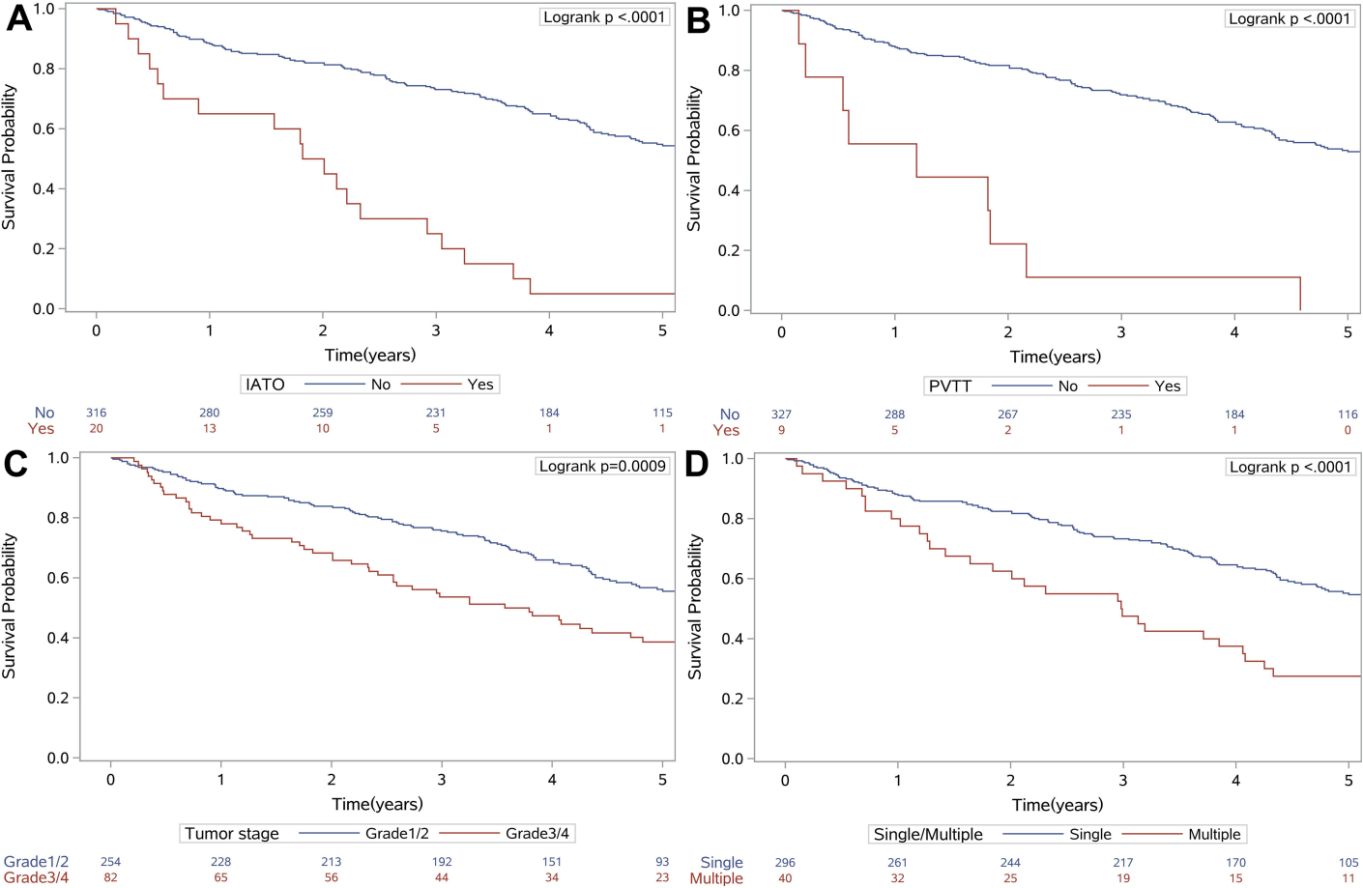
Kaplan-Meier survival curves for IATO (**A**), PVTT (**B**), tumor stage (**C**) and single/multiple tumors (**D**), which were independently associated with mortality. Abbreviations: IATO: invasion of adjacent tissues or organs; PVTT: portal vein tumor thrombus.

As tumor size, PT, and AST were continuous variables that were associated with mortality, we aimed to identify the optimal cut-off values using X-tile analysis, which is an effective tool for evaluating biomarkers’ and other factors’ abilities to predict patient survival. To the best of our knowledge, this is the first study to examine the optimal prognostic cut-off values for these three variables using X-tile analysis. The results are shown in Figure 1, which indicated that the optimal cut-off values were 3.0 cm for tumor size, 14.5 s for PT, and 65.0 U/L for AST. Figure 2 shows the Kaplan-Meier curves for predicting mortality based on IATO, PVTT, tumor stage and single/multiple tumors. The results indicate that those factors were strong predictors of mortality. As Figure 2 was shown, the cumulative mortality rates were 95.0% and 43.0% in the population with and without IATO as well as 100.0% and 44.6% in the population with PVTT and without PVTT, respectively. In addition, the corresponding mortality rates were 41.7% in the Grade1/2 tumor group and 59.8% in the Grade3/4 tumor group, as well as 42.6% for single tumor and 72.5% for multi tumor.

### Development of the RSS

The seven independent predictors from the previous section were used to create an RSS, based on their beta regression coefficients (Table 2). These scores were calculated using linear transformations of the corresponding beta coefficients (divided by 0.37, the minimum beta value for tumor stage), multiplication by a constant value of 2, and rounding to the nearest integer. The reference groups for the categorical variables were assigned scores of 0, based on a beta coefficient of zero [18]. The total risk score was then calculated using the following formula:

$$\begin{array}{l} Prognostic\;score=2\times [grade\;3\text{/}4\;tumor]\\ \;\;+5\times [tumor\;size\;of>3.0\;cm]+5\times [PVTT\;present]\\ \;\;+5\times [PT\;of>14.5\;s]+3\times [multiple\;tumors\;]\\ \;\;+6\times [IATO\;present]+2\times [AST\;of>65.0\;U\text{/}L]. \end{array}$$

**Table 2:** Multivariate Cox proportional-hazards analysis of the development cohort and scoring system.

**Risk factors**	**HR(95%CI)**	**P^¶^**	**β regression Coefficient**	**Point**
Grade ¾ tumor	1.450(1.025,2.053)	0.036	0.37	2
Tumor size (>3.0cm)	2.616(1.808,3.786)	<0.001	0.96	5
PVTT	2.439(1.185,5.021)	0.016	0.89	5
PT (>14.5s)	2.480(1.754,3.507)	<0.001	0.91	5
Multiple tumors	1.922(1.270,2.909)	0.002	0.65	3
IATO	3.318(2.011,5.474)	<0.001	1.20	6
AST (>65.0U/L)	1.488(1.061,2.086)	0.021	0.40	2

**Note:** Weighted points of a risk factor were calculated using a linear transformation of the corresponding β coefficient (was divided by 0.37 [the lowest β value of individual variable, the Tumor stage], multiplied by a constant (2), and rounded to the nearest integer). The reference group of categorical variables was assigned 0 point, corresponding to a beta coefficient of zero.

**Abbreviations:** PT: prothrombin time; PVTT: portal vein tumor thrombus; IATO: invasion of adjacent tissues or organs. AST: aspartate transaminase;

¶:P values are for the comparison of corresponding reference group with patients in Categorical variables (%).

**Figure 3:**
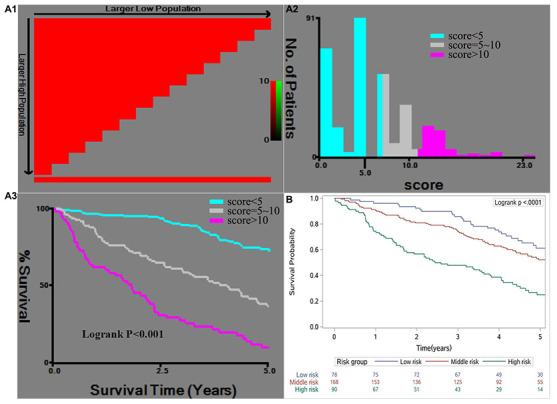
X-tile analysis of the risk score in the training cohort (**A1**, **A2**, **A3**) and the Kaplan-Meier survival curves for the risk groups in the validation cohort (**B**). The cut-off points for the risk groups were determined based on A2 as <5 (low risk), 5–10 (medium risk), and >10 (high risk).

In the training cohort, the total scores were subsequently classified as <5 (low risk of mortality), 5–10 (medium risk of mortality), and >10 (high risk of mortality) (Figure 3, A1 and A2). The cumulative probabilities of OS in the risk groupings are shown in Figure 3 (A3).

### Validation of the RSS

**Figure 4:**
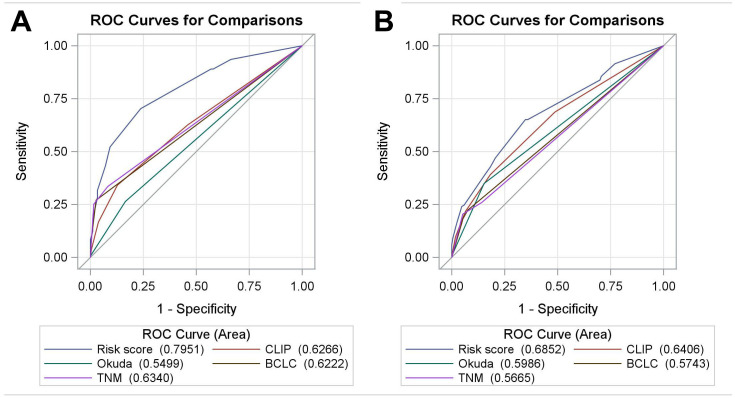
The receiver operating characteristic curves for the RSS, CLIP, BCLC, Okuda, and TNM systems in the training cohort (**A**) and the validation cohort (**B**). Abbreviations: BCLC: Barcelona Clinic Liver Cancer; CLIP: Cancer of the Liver Italian Program; RSS: risk scoring system.

Total prognostic scores were then calculated in the validation cohort base on the RSS. The Kaplan-Meier curves of validation cohort between risk groups (*P*<0.001), were similar to training cohort, showed that the RSS was robust in predict OS (Figure 3B). The effectiveness and accuracy of the RSS was evaluated by comparing it to various other widely used systems in the training and validation cohorts (Figure 4). Relative to the CLIP, BCLC, TNM, and Okuda systems, the RSS provided the strongest ability to predict mortality in PHCC cases based on AUC values of 0.80 in the training cohort and 0.69 in the validation cohort. In addition, we examined the proportions of the patients assigned to the risk groupings in the validation cohort (low risk: 23.2%, medium risk: 50.0%, high risk: 26.8%), which were similar to the proportions from the training cohort (low risk: 28.3%, medium risk: 47.9%, high risk: 23.8%). Relative to the low-risk group, the high-risk group had elevated risks of mortality in the training cohort (HR: 9.681, 95% CI: 5.664–16.549) and in the validation cohort (HR: 3.211, 95% CI: 2.045–5.042). Moreover, we determined the differences in the risk of mortality between the low-risk and high-risk groups, which were determined to be 65.9% in the training cohort and 36.5% in the validation cohort. This result indicated that the RSS had excellent discriminating ability, and the Hosmer-Lemeshow test revealed good calibration in the training and validation cohorts (both *P*>0.05). Finally, the C-index value of 0.663 (95% CI: 0.618–0.708) in the validation cohort indicated that the RSS had excellent discrimination and good calibration (Table 3).

**Table 3:** Mortality by risk group in patients for all variables of the multiple logistic regression model in the development and validation cohorts.

	**Development cohort(N=336)**				**Validation cohort(N=336)**			
	**N (%)**	**Death (%)•**	**HR (95%CI)**	**P**	**N (%)**	**Death (%)**	**HR (95%CI)**	**P**
Risk group								
Low-risk (<5)	95(28.3)	17(17.9)	Ref	Ref	78(23.2)	27(34.6)	Ref	Ref
Medium-risk (5~10)	161(47.9)	71(44.1)	2.905(1.711,4.932)	<0.001	168(50.0)	75(44.6)	1.433(0.923,2.224)	0.109
High-risk (>10)	80(23.8)	67(83.8)	9.681(5.664,16.549)	<0.001	90(26.8)	64(71.1)	3.211(2.045,5.042)	<0.001
Risk score performance AUC(95% CI)	0.80(0.75,0.84)		0.69(0.63,0.74)	
Hosmer–Lemeshow goodness of fit test**§**	Chi-square = 3.532 (P = 0.618)				Chi-square = 7.719 (P = 0.172)			
C-index(95%CI)	0.741(0.695,0.787)				0.663(0.618,0.708)			
Difference in mortality**ξ**	65.9%				36.5%			

£: Mortality of each risk group.

§:Non-significant Hosmer–Lemeshow goodness of fit test indicates good calibration.

ξ:Difference in mortality between high-risk and low-risk groups (%), calculated by [mortality in high-risk group] - [mortality in low-risk group].

Abbreviations: HR: Hazard ratio; CI: Confidence incidence; C-index: Concordance-index.

## DISCUSSION

The most common primary liver cancer is PHCC, which is also the main cause of death in people with cirrhosis [16]^.^ However, relative to the previous decades, recent therapeutic improvements have enhanced the survival of current PHCC patients, who receive effective treatment using surgical resection, ablation, trans-arterial chemoembolization, and hepatic arterial infusion chemotherapy plus sorafenib [19, 20]. Hepatectomy is widely recognized as a treatment for select HCC patients, in whom it provides similar long-term outcomes among patients with both early-stage and advanced disease [21, 22]. Although various prognostic scores have been developed for PHCC [23, 24], these scores have not been accepted by consensus as a standardized system [25, 26] for predicting outcomes in this setting, some internationally recognized and widely used systems were also not satisfactory in terms of predicting OS. Thus, predicting the postoperative survival of PHCC patients remains a challenge for clinicians, and it would be useful to have a reliable scoring system that was based on one or several biomarkers and clinical factors. This study developed and validated an RSS for predicting OS after resection of PHCC, and it appears that the simple RSS may be a useful tool for clinicians and medical staff to identify patients with increased mortality risk, who may require personalized management strategies. 2 main findings were summarized as follows: First, we report a negative correlation between OS of PHCC after resection and common clinical or laboratory parameters, which are higher tumor stage, multiple tumors, PVTT, IATO, greater tumor size, and elevated values for PT and AST. Second, A risk score were calculated by above risk factors using their corresponding beta coefficients, the RSS results were then classified as three risk groups including low, medium and higher risk for predicting OS.

In this context, previous studies have indicated that tumor stage, tumor size, and multiple tumors were strong predictors of poor OS in PHCC cases [27–29]. The present study was also emphasized the importance of tumor burden consistent with those findings and identified the optimal cut-off value as 3.0 cm of tumor size based on the X-tile analysis, as well as optimal cut-off values for PT and AST, which are known markers of liver failure based on previous studies [30, 31]. Thus, we believe that these useful thresholds, which were determined using a robust statistical approach, may enhance the clinical utility of the RSS. Mizumoto et al. and Zhang et al. [32, 33] have also reported that the absence of PVTT was associated with good survival in HCC cases, and our findings support this relationship by indicating that the presence of PVTT predicted a poor prognosis in PHCC cases. Previous research has also indicated that a tumor’s invasion of adjacent and distant tissues is a risk factor for mortality [34], which explains why IATO was a significant risk factor for mortality in our PHCC cases. Interestingly, our RSS did not consider various clinical factors (e.g., age and sex) that are reportedly associated with mortality in PHCC cases [35]. This may be related to the laboratory parameters being more sensitive for predicting mortality, which would result in the clinical factors being omitted from the multivariable model.

The RSS are more accurate than traditional staging systems in predicting the prognosis of PHCC. The advantage is that the RSS is easy to calculate based on common clinical and laboratory parameters. Moreover, the accuracy comparison of AUC curve showed that the RSS predicted the best OS after curative liver resection than the CLIP, BCLC, TNM, and Okuda systems in the training cohort. Because we intended the RSS to help guide personalized treatment of high-risk PHCC cases, we categorized the risk scores into three groups (high, medium, and low risk). Using this approach may allow for customized approaches to managing these risk groups, which may improve the cost-effectiveness of their treatment. We also validated the RSS using the validation cohort, which confirmed that the RSS had better prognostic performance than the CLIP, BCLC, TNM and Okuda systems. These results suggest that the RSS may be clinically useful, robust, effective, and accurate for predicting mortality in PHCC cases.

The present study has two important limitations. First, the retrospective single-center design is associated with a risk of selection bias, as well as decreased statistical efficiency and testing power. Second, the patients were all from the same region of China (Wenzhou), and the results may not be generalized to other areas or ethnicities. Therefore, additional external verification is needed to confirm whether our RSS is useful in other regions and patient populations.

In conclusion, the present study revealed that an RSS could effectively predict OS in PHCC cases, and that may help clinicians and medical staff to improve patients’ quality of life after resection of PHCC.

## MATERIALS AND METHODS

### Research design and data sources

This retrospective study evaluated 672 patients who underwent resection of PHCC at our hospital between January 2007 and February 2015. Face-to-face interviews had been performed before the operation to collect data regarding sex, history of alcohol abuse, age, height, weight, and body mass index (BMI, kg/m^2^). Preoperative blood tests had also been routinely performed to collect data regarding alpha fetoprotein (AFP) levels, prothrombin time (PT), fibrinogen (FIB) levels, neutrophil counts, monocyte counts, lymphocyte counts, platelet (PLT) counts, albumin (ALB) levels, total bilirubin (TBIL) levels, total cholesterol (TC) levels, aspartate transaminase (AST) levels, γ-glutamyl transpeptidase (γ-GT) levels, and alanine transaminase (ALT) levels. Surgical records were searched to collect data regarding the pathological diagnosis, ascites, cirrhosis, tumor size, tumor capsule, single or multiple tumors, satellite nodules, degree of tumor differentiation, peri-cancerous invasion, bile duct infiltration, lymph node metastasis (LNM), microvascular invasion (MVI), nerve infiltration, portal vein tumor thrombus (PVTT), intrahepatic metastases, and invasion of adjacent tissues or organs (IATO). The date of the hepatectomy was defined as the start of follow-up and the final outcome was recorded based on the date of death or the last known status on August 1, 2018. Strict data quality control measures were performed using double verification before the data analysis. The research was approved by our hospital ethics committee. Patient consent was obtained by telephone.

### Patients and testing methods

The inclusion criteria were: 1) complete blood test data from before the surgery; 2) a postoperative pathological diagnosis of PHCC; 3) complete tumor resection during the surgery; 4) no preoperative cancer treatment; 5) no history of other malignant tumors or tumor-related complications; 6) good heart, brain, and kidney functioning before the surgery; and 7) no severe postoperative complications, such as massive bleeding or liver failure. Detailed information indicates the reasons for the number of patients screened excluded and included can be seen in Supplementary Figure 1. Based on these criteria, we excluded 380 PHCC cases among 1052 PHCC patients in the present research. In the follow-up period, we finally observed 321 dead PHCC patients as the case group and 351 alive PHCC patients as the control group in this nested case-control study. After that, we identified 672 PHCC cases that were retrospectively randomized in a 1:1 ratio to the training cohort (N=336) and the validation cohort (N=336).

All patients underwent routine blood collection before surgery and the samples were sent to a laboratory for analysis. Tumor marker levels (ie, AFP levels) were determined using the Unicel DXI-800 Analyzer (Beckman Coulter Inc., Japan). Markers of coagulation function (eg, PT, FIB levels, and other factors) were measured using a STA-R automated coagulation analyzer (French diagnostic criteria). Blood biochemical indicators (eg, TBIL, ALT, and AST) were measured using a Beckman AU5800 automatic biochemical analyzer (Beckman Coulter Inc., Japan). Routine testing for blood components (eg, PLT, lymphocyte, and neutrophil counts) was performed using a SysMex XE-2100 automated blood cell analyzer (SysMex Corporation, Japan). All testing methods were performed as previously described [8]. The tumor samples were re-analyzed and the pathological diagnosis was confirmed by the pathology department at our hospital.

### Statistical analysis

Categorical variables were reported as number (percentage), while continuous variables were reported as median (interquartile range [IQR]) based on the apparently skewed distributions. Depending on the training set, we performed univariate and multivariable Cox proportion hazard regression models to screen potential prognostic factors. According to the Akaike information criterion (AIC), we fitted a series of different multivariable models, factors with p-values over 0.05 in the univariate Cox proportion hazard regression models would be removed from the multivariable Cox proportion hazard regression model. Optimal cut-off values for the independent continuous variables were identified using X-tile analysis in the training cohort. Kaplan-Meier curves and the log-rank test were used to identify differences in survival probability.

Independent risk factors identified using the multivariate Cox proportional hazards model were assigned risk scores based on their β regression coefficient. The total risk score was then calculated for each patient by adding together the individual risk scores for each applicable risk factor. The discriminative ability of the RSS was estimated using the Hosmer-Lemeshow goodness of fit test and the concordance index (C-index) in the training and validation cohorts. Receiver operating characteristic (ROC) curves were used to compare the predictive abilities of the RSS and various traditional prognostic models.

All tests were two-sided and *P*-values of ≤0.05 were considered statistically significant. All data management and statistical analyses were performed using IBM SPSS Statistics software (version 22.0; IBM Corp., Armonk, NY). Figures were created using R-studio for Windows (version 1.1.456, copyright 2009–2018; RStudio Inc.) and X-tile software (Robert L Camp, MD, PhD; copyright Yale University 2003–2005).

### Data availability

All study-related data are included in the published report.

## Supplementary Material

Supplementary Figure 1

Supplementary Table 1
