# Supplementary figures and images for: Development and validation of a risk score for predicting mortality after resection of primary hepatocellular carcinoma
Source: Aging (Albany NY). 2020 Jun 21;12(12):11878–92. doi: 10.18632/aging.103360 (PMC7343477; doi:10.18632/aging.103360)

## SUPPLEMENTARY FIGURE

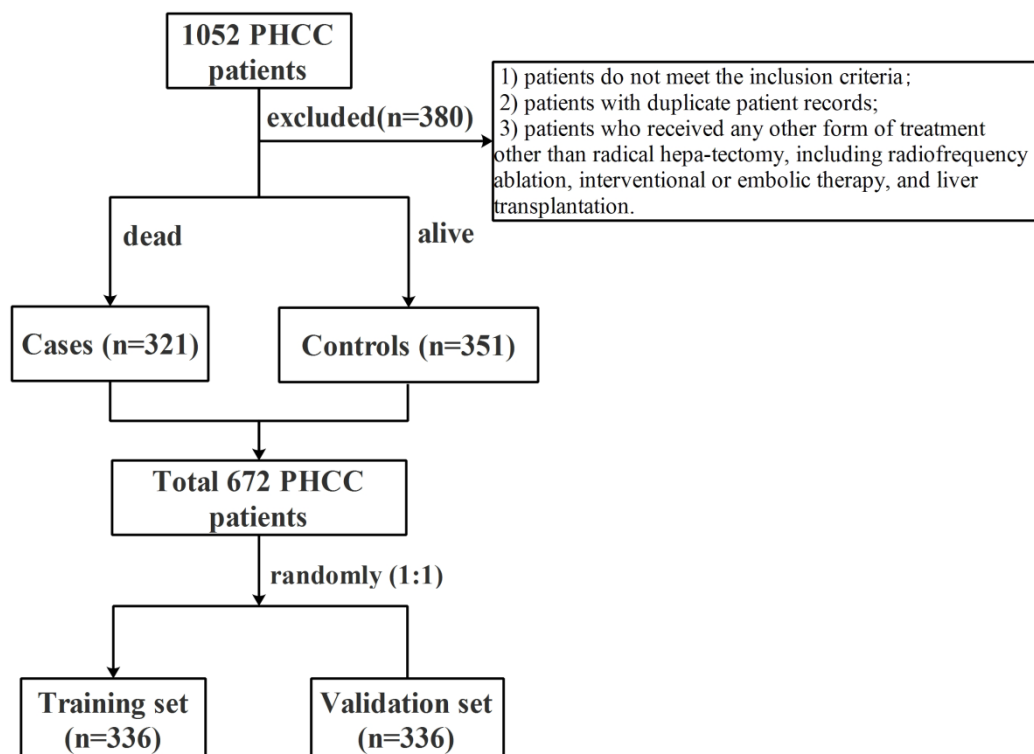

Supplementary Figure 1. Study flowchart of PHCC cases chosen.

Supplement: Supplementary Figure 1 [file aging-12-103360-s001..pdf]
