# Supplementary material for: Development and validation of a risk score for predicting mortality after resection of primary hepatocellular carcinoma
Source: Aging (Albany NY). 2020 Jun 21;12(12):11878–92. doi: 10.18632/aging.103360 (PMC7343477; doi:10.18632/aging.103360)
Supplement: Supplementary Table 1 [file aging-12-103360-s002..pdf]

SUPPLEMENTARY TABLE

Supplementary Table 1. Baseline demographics and clinical characteristics of patients in training cohort and validation cohort.

Variables	Training set N=336	Validation set N=336	*P*-value
**Continuous variables**			
Age(years)	57.0(49.0,63.0)	57.0(48.5,64.0)	0.612
Height(cm)	168.0(160.5,170.0)	168.0(162.0,170.0)	0.730
Weight(kg)	62.0(56.0,70.0)	63.0(56.8,68.0)	0.373
BMI (kg/m^2)	22.8(20.8,24.8)	22.2(20.6,24.1)	0.057
Tumor size(cm)	3.5(2.5,5.0)	4.0(2.5,6.0)	0.031
PT (seconds)	13.9(13.3,14.7)	13.9(13.3,14.8)	0.650
FIB(g/L)	2.8(2.4,3.4)	2.8(2.4,3.6)	0.388
Neutrophil (x10^9/L)	3.2(2.4,4.2)	3.2(2.4,4.3)	0.933
Monocyte (x10^9/L)	0.4(0.3,0.6)	0.4(0.3,0.6)	0.241
Lymphocyte (x10^9/L)	1.4(1.0,1.8)	1.4(1.1,1.8)	0.945
Platelet (x10^9/L)	136.0(94.5,171.0)	147.0(100.0,191.0)	0.026
ALB (g/L)	39.7(36.3,43.0)	40.0(35.9,43.3)	0.761
TBIL(μmol/L)	11.0(8.0,16.0)	11.0(8.0,16.0)	0.519
TC (mmol/l)	4.3(3.5,4.9)	4.3(3.7,4.9)	0.212
ALT(U/L)	34.0(24.0,54.5)	35.0(22.5,50.5)	0.466
AST(U/L)	40.5(30.0,68.0)	37.0(28.0,60.0)	0.158
γ-GT(U/L)	54.0(34.0,106.0)	50.5(31.5,95.5)	0.295
**Discrete variables**			
Sex			1.000
Man	280(83.3)	280(83.3)	
Woman	56(16.7)	56(16.7)	
History of alcohol abuse			0.060
No	185(55.1)	209(62.2)	
Yes	151(44.9)	127(37.8)	
Nerve infiltration			1.000
No	336(100.0)	335(99.7)	
Yes	0(0.0)	1(0.3)	
Bile duct infiltration			0.808
No	299(89.0)	297(88.4)	
Yes	37(11.0)	39(11.6)	
LNM			0.401
No	328(97.6)	331(98.5)	
Yes	8(2.4)	5(1.5)	
Tumor grade			0.139
Grade1/2	254(75.6)	237(70.5)	
Grade3/4	82(24.4)	99(29.5)	
Peri-cancerous invasion			0.406
No	322(95.8)	326(97.0)	
Yes	14(4.2)	10(3.0)	
Intrahepatic metastasis			0.682
No	334(99.4)	332(98.8)	
Yes	2(0.6)	4(1.2)	
Tumor capsule			0.576
No	265(78.9)	259(77.1)	
Yes	71(21.1)	77(22.9)	
Satellite nodules			0.636
No	313(93.2)	316(94.0)	
Yes	23(6.8)	20(6.0)	

Single/multiple			0.810
No	296(88.1)	298(88.7)	
Yes	40(11.9)	38(11.3)	
PVTT			0.154
No	327(97.3)	320(95.2)	
Yes	9(2.7)	16(4.8)	
Vascular infiltration			0.270
No	318(94.6)	311(92.6)	
Yes	18(5.4)	25(7.4)	
IATO			0.211
No	316(94.0)	323(96.1)	
Yes	20(6.0)	13(3.9)	
Cirrhosis			0.070
No	98(29.2)	120(35.7)	
Yes	238(70.8)	216(64.3)	
Ascites			0.674
No	284(84.5)	280(83.3)	
Yes	52(15.5)	56(16.7)	
HBsAg			0.098
Negative	57(17.0)	74(22.0)	
Positive	279(83.0)	262(78.0)	
AFP (ug/L)			0.124
<400	264(78.6)	247(73.5)	
≥400	72(21.4)	89(26.5)	
TNM stage			0.984
I	269(80.1)	269(80.1)	
II	25(7.4)	24(7.1)	
III/IV	42(12.5)	43(12.8)	
Child-Pugh class			0.756
A	233(69.3)	228(67.9)	
B	101(30.1)	107(31.8)	
C	2(0.6)	1(0.3)	
CLIP			0.757
0	156(46.4)	139(41.4)	
1	104(31.0)	101(30.1)	
2	43(12.8)	49(14.6)	
3	26(7.7)	28(8.3)	
4	7(2.1)	18(5.4)	
5	0(0.0)	1(0.3)	
Okuda			0.247
I	265(78.9)	252(75.0)	
II	70(20.8)	80(23.8)	
III	1(0.3)	4(1.2)	
BCLC			0.805
A	289(86.0)	286(85.1)	
B	13(3.9)	11(3.3)	
C	32(9.5)	35(10.4)	
D	2(0.6)	4(1.2)	

Abbreviations: BMI: body mass index; FIB, fibrinogen; ALB, albumin; PT: prothrombin time; TBIL: total bilirubin; TC: total cholesterol; ALT: alanine transaminase; AST: aspartate transaminase; γ-GT: γ-glutamyl transpeptidase; LNM: lymph node metastasis; PVTT: portal vein tumor thrombus; IATO: invasion of adjacent tissues or organs; AFP: alpha fetoprotein; TNM: Tumor–Node–Metastasis; CLIP: Cancer of the Liver Italian Program; BCLC: Barcelona Clinic Liver Cancer.
